# Optimization of Solid-State Fermentation Process for Prodigiosin Production from Soybean Residue Using *Serratia marcescens* BD2025 and Evaluation of Pigment Properties

**DOI:** 10.3390/microorganisms14071475

**Published:** 2026-07-06

**Authors:** Ting Yang, Wenlu Bi, Rui Zhang, Qianqian Jia, Yu Wang, Deping Han, Jiahui Han, Haojie Sha, Zhanqiang Ma, Dingding Su

**Affiliations:** Peking University Institute of Advanced Agricultural Sciences, Shandong Laboratory of Advanced Agricultural Sciences in Weifang, Weifang 261325, China

**Keywords:** soybean residue, *Serratia marcescens* BD2025, prodigiosin, condition optimization, stability, antibacterial, antioxidant activity

## Abstract

Soybean residue, a major by-product of soy milk and tofu processing, is a large-yield and nutrient-rich substrate, but it is currently experiencing low utilization rates. In this study, a prodigiosin-producing strain, *Serratia marcescens* BD2025, was isolated from naturally fermented soybean residue. Subsequently, prodigiosin was produced by solid-state fermentation of soybean residue, and the fermentation conditions were further optimized by a Box–Behnken (BBD) model involving the soybean residue-to-water ratio, inoculum size, and temperature. Under the optimized conditions of a soybean residue-to-water ratio of 1:4.28, an inoculum size of 5.2%, and cultivation at 27.5 °C for 48 h, the prodigiosin yield reached 19.05 mg/g. High-performance liquid chromatography analysis showed that the major pigment peak had a retention time comparable to that of a prodigiosin standard, with an estimated purity of 96% based on peak area normalization. The results show that prodigiosin remains relatively stable at below 40 °C, in acidic conditions, and in darkness. The extracted prodigiosin inhibited both *Escherichia coli* and *Staphylococcus aureus*, with stronger activity against *S. aureus*. The scavenging ability of DPPH radical was dose-dependent, with the scavenging activity of 93.13% at 5 mg/mL. These findings indicate that soybean residue can serve as a solid-state fermentation substrate for prodigiosin production and provide a basis for the value-added utilization of soybean-processing residues.

## 1. Introduction

Prodigiosin is a red secondary metabolite composed of a tricyclic pyrrole ring [1]. It has been reported to exhibit antibacterial [2], anti-cancer [3,4], immunosuppressive [5], insecticidal [6], antimalarial [7], and antiparasitic activities [8]. These biological properties have supported its potential application in food, pharmaceutical, cosmetic, textile, and biomaterial-related fields [9]. Prodigiosin can be produced by chemical synthesis or microbial fermentation [10]. However, the chemical synthesis method has several drawbacks, including poor system stability, easy formation of isomers, difficult control of the synthesis process, and high production costs [1]. In contrast, microbial fermentation provides a more sustainable production route because it can be conducted under mild conditions, reduces dependence on chemical synthesis steps, and generates biologically derived pigments with high purity [11]. Accordingly, microbial fermentation has become one of the most promising strategies for prodigiosin production. Microorganisms are the principal natural sources of prodigiosin, although pigment yield varies substantially among species, strains, and cultivation condition [12]. Among prodigiosin-producing bacteria, *Serratia marcescens* (*S. marcescens*) is one of the most extensively investigated species [13]. *S. marcescens* is a Gram-negative bacterium that is widely distributed in humid environments, including ecosystems and hospital environments [14]. Pigment production by *S. marcescens* is strongly affected by environmental and nutritional factors, particularly temperature, pH, carbon source, nitrogen source, and medium composition [15]. Previous studies have reported that prodigiosin production by *S. marcescens* is generally favored at approximately 20–28 °C [15]. Deviations from this temperature range can markedly reduce pigment formation [16]. Therefore, optimization of both medium formulation and fermentation conditions is essential for improving prodigiosin yield.

Prodigiosin not only demonstrates strong biological activity but also shows unique potential in the development of new materials, such as intelligent color-changing packaging, anti-MRSA wound dressings and fabric dyeing [17]. Therefore, the demand for prodigiosin production is increasing. Despite its biological and industrial potential, the large-scale production of prodigiosin remains limited by the cost of conventional nutrient media [18]. This limitation has stimulated interest in low-cost agro-industrial residues and food-processing by-products as alternative substrates for microbial pigment production. Several waste-derived materials have been evaluated for prodigiosin fermentation. For example, sugarcane bagasse has been used as a solid-state fermentation substrate for *S. marcescens* Xd-1, achieving a prodigiosin yield of 79 mg/kg dry solid [19]. Fermentation of *S. marcescens* SBL03 using agricultural and industrial wastes increased prodigiosin production by 7.62-fold [20]. Soybean meal has also been used as the sole nutritional source for *S. marcescens*, resulting in a 5.8-fold higher yield than that obtained with commercial media [21]. In addition, *S. marcescens* NCHU05 produced significantly higher levels of prodigiosin when food waste was used as the fermentation medium than when conventional liquid medium was used [22]. These studies demonstrate that waste-derived substrates can support prodigiosin biosynthesis and may improve the economic and environmental sustainability of microbial pigment production.

Solid-state fermentation (SSF) is particularly suitable for the bioconversion of solid organic residues because it operates with limited free water, can reduce contamination risk, lowers liquid effluent generation, and facilitates the handling or reuse of spent fermentation materials [23]. Compared with submerged fermentation, solid-state fermentation may better match the physical characteristics of many agro-industrial by-products and food-processing residues. Therefore, integrating solid-state fermentation with nutrient-rich waste substrates provides a practical strategy for producing high-value microbial metabolites while promoting waste valorization.

Soybean residue is a major by-product of soymilk and tofu processing, with annual global production estimated at millions of tons [24]. Owing to its high contents of dietary fiber, protein, isoflavones and minerals, soybean residue represents a nutrient-rich organic feedstock rather than merely a processing waste [25]. However, its high moisture content, rapid spoilage, and limited economic value in conventional applications have restricted its large-scale utilization, and a considerable proportion is still directed to low-value uses or remains underutilized [26]. The valorization of soybean-processing by-products has therefore attracted increasing attention as a strategy for developing functional ingredients and microbial bioprocesses [27]. In particular, the protein and carbohydrate-rich composition of soybean residue makes it a suitable substrate for microbial growth and metabolite production [25]. Previous studies have demonstrated its use in protease production [28] and probiotic cultivation, including lactic acid bacteria fermentation [29]. Nevertheless, the direct use of soybean residue as a solid-state fermentation substrate for prodigiosin production by *S. marcescens* has not been systematically investigated. Developing such a process could provide a value-added route for soybean residue utilization while reducing the medium cost associated with microbial pigment production.

In this study, soybean residue was investigated as a low-cost substrate for solid-state prodigiosin production by *Serratia marcescens* BD2025, a strain isolated from naturally fermented soybean residue. Red pigment was produced using bean residue as the solid-state fermentation substrate. The study focused on strain isolation and identification, pigment extraction and characterization, optimization of fermentation conditions using a Box–Behnken design, and evaluation of pigment stability, antibacterial activity, and DPPH radical scavenging activity. The objective was to establish a sustainable fermentation strategy for converting soybean-processing residue into a high-value microbial pigment. The findings may provide a basis for the development of waste-based bioprocesses for prodigiosin production and support the value-added utilization of soybean residue.

## 2. Materials and Methods

### 2.1. Isolation and Identification of S. marcescens BD2025

The soybean residue used in this article was provided by Yuwang Group in Dezhou, Shandong, China. A red pigment-producing strain was isolated from naturally fermented soybean residue in nature. Briefly, 1.0 g of fermented soybean residue was transferred into 10 mL of sterile physiological saline. The resulting suspension was serially diluted with sterile physiological saline, and appropriate dilutions were spread onto nutrient agar (NA) plates. The plates were incubated at 28 °C for 48 h. Red-pigmented colonies were selected and repeatedly streaked on NA plates until morphologically uniform colonies were obtained. The purified isolate was designated BD2025. The bacterial strain DNA was extracted and purified using the DNA extraction kit (Bao Biotechnology Engineering Co., Ltd., Dalian, China). The integrity of the DNA was checked using a nucleic acid electrophoresis instrument (SC12, Beijing Kaiyuan Xinxie Instrument Co., Ltd., Beijing, China). The 16S rDNA fragment was amplified using the universal primers 27F (5′–AGA GTT TGA TCC TGG CTC AG–3′) and 1492R (5′–ACG GTT ACC TTG TTA CGA CTT–3′). The reaction system is as follows: 2 μL template DNA, 1 μL 27F primer, 1 μL 1492R primer, 12.5 μL Taq DNA polymerase, and 8.5 μL ddH_2_O. The reaction conditions were 98 °C for 5 min, 95 °C for 35 s, 55 °C for 35 s, 72 °C for 90 s, and 35 cycles for 8 min. After the reaction, the PCR products were detected by 1% agarose gel electrophoresis. Then, the PCR products were subjected to DNA sequencing. The results were submitted to the National Center for Biotechnology Information (NCBI) and subjected to an online comparison using the basic local alignment search tool (BLAST, https://blast.ncbi.nlm.nih.gov/Blast.cgi, access date is: 16 June 2026). Subsequently, a phylogenetic tree was drawn using the MEGA-X software (v. 10.2.6). Based on sequence analysis, the isolate was identified as *Serratia marcescens* BD2025.

### 2.2. Preparation and Analysis of Prodigiosin

Prodigiosin extraction and quantification were carried out according to the method described by Gohil et al. [21], with modifications. *S. marcescens* BD2025 was cultured in nutrient broth medium to the logarithmic phase. Then, 5% of the bacterial solution was taken and inoculated into the cooled and sterilized soybean residue. Solid-state fermentation was carried out at 28 °C for 48 h. The pigment was extracted from the soybean residue using ethanol (analytical-grade, Sinopharm Chemical Reagent Co., Ltd., Shanghai, China) as the solvent at a solid-to-liquid ratio of 1:10 (*w*/*v*). The mixture was treated with an ultrasonic cleaning machine (40 kHz, 720 W, Ningbo Xinzhi Biotechnology Co., Ltd., Ningbo, China) at 28 °C for 30 min. The extract was centrifuged at 12,000 rpm for 10 min at 4 °C, and the supernatant was collected. The extraction was repeated until the solid residue became nearly colorless. The combined supernatants were filtered through a 0.22 µm PES syringe filter (FilterBio PES Syringe Filter, Lab-Ex Ltd., Budapest, Hungary) and concentrated under reduced pressure using a rotary evaporator (Rotavapor RV.10, IKA Staufen, Staufen, Germany). The concentrate was subsequently lyophilized for 72 h using a freeze dryer (LaboGene, Lillerød, Denmark) to obtain crude pigment powder.

The extracted pigment was analyzed using an Agilent 1260 Infinity II high-performance liquid chromatograph (HPLC, Agilent Technologies, San Jose, CA, USA), and its relative purity was estimated using the peak area normalization method [30]. A prodigiosin standard and the extracted pigment sample were analyzed under the same chromatographic conditions. The pigment extraction was detected using a diode array detector (DAD). Separation was performed using an Agilent Zorbax SB-C18 column (4.6 × 250 mm, 5 µm). The mobile phase consisted of acetonitrile (HPLC gradient grade, ≥99.9%, Merck, Darmstadt, Germany) and 5% aqueous ammonium acetate solution (10 mM, pH 5.0) at a ratio of 85:15 (*v*/*v*), with isocratic elution at a flow rate of 1.0 mL/min. The column temperature was maintained at 40 °C, the injection volume was 10 µL, and the total run time was 10 min. Detection was performed at 535 nm.

### 2.3. Optimization of Prodigiosin Fermentation Process Based on Box-Behnken Design

The optimization procedure was adapted from Gohil et al. [20] to improve prodigiosin production from soybean residue. Response surface methodology (RSM) was used to optimize prodigiosin production conditions, a Box–Behnken design (BBD) was used to fit the model by least squares, and the proposed model was evaluated by variance analysis (ANOVA). In this study, the soybean residue-to-water ratio (X1), the inoculation size (X2), and cultivation temperature (X3) were selected as independent variables, and prodigiosin yield was used as the response variable. The coded levels of the independent variables are shown in Table 1.

A total of 17 experimental runs, including five center-point replicates, were performed according to the BBD matrix shown in Table 2. All fermentations were conducted for 48 h. A second-order polynomial model was fitted to the experimental data by least-squares regression. Model significance, lack of fit, and the effects of individual and interactive variables were evaluated by analysis of variance (ANOVA) using Design-Expert 13 software. Three-dimensional response surface plots and contour plots were generated to visualize the interactions among the selected variables and to determine the optimal fermentation conditions.

### 2.4. Stability Analysis of Prodigiosin

The stability of prodigiosin was determined using the method described by Shuhua Liu et al. [31], with some minor modifications. A methanol (analytical grade, Sinopharm Chemical Reagent Co., Ltd.) solution of 1 mg/mL of prodigiosin was prepared, and its initial absorbance value was measured at 535 nm. Equal-volume aliquots of the prodigiosin solution were then subjected to different temperature, pH, and light treatments. For thermal stability analysis, aliquots were incubated in the dark at 20, 40, 60, 80, and 100 °C, and the absorbance at 535 nm was measured at 1 h intervals. For pH stability analysis, aliquots were adjusted to pH 1, 3, 5, 7, 9, 11, and 13, and absorbance was measured at 4 h intervals in the dark. For light stability analysis, aliquots were exposed either to darkness or natural light, and absorbance was measured at 2 h intervals. The pigment retention content was calculated using the following equation:

Residual pigment content (%) = A_t_/A_0_ × 100

where A_0_ is the initial absorbance, and A_t_ is the absorbance after treatment.

### 2.5. Evaluation of Antibacterial Activity of Prodigiosin In Vitro

The antibacterial activity of prodigiosin was evaluated by using an agar diffusion assay according to the method described by Xin Wang et al. [32], with modifications. The specific operation is as follows: *Staphylococcus aureus* (*S. aureus*, ATCC 6538, Beijing Soleibao Biotechnology Co., Ltd., Beijing, China) and *Escherichia coli* (*E. coli*, ATCC 8099, Beijing Soleibao Biotechnology Co., Ltd., Beijing, China) were prepared into bacterial suspension, and 0.2 mL of each suspension was evenly spread on the plates. Sterile filter paper discs (6 mm in diameter) were immersed for 30 min in ethanolic prodigiosin solutions at concentrations of 0, 10, 40, 160, and 640 µg/mL. The 0 µg/mL treatment served as the solvent control. After the bacterial solution had been absorbed into the agar surface, the plates were incubated at 30 °C for 18 h. The diameters of the inhibition zones were measured after incubation. Each concentration was tested in triplicate, and the results were expressed as the mean inhibition-zone diameter.

### 2.6. DPPH Radical Scavenging Ability

The antioxidant activity of prodigiosin was assessed using the 1,1-Diphenyl-2-picrylhydrazyl (DPPH) radical scavenging assay according to Ibrahim et al. [33], with modifications. The assay was performed using an DPPH free-radical scavenging kit (Beijing Solarbio Science & Technology Co., Ltd., Beijing, China). Briefly, prodigiosin sample solution was mixed with 950 µL of DPPH working solution. The mixture was incubated at room temperature in the dark for 30 min, and the absorbance was immediately measured at 517 nm. Sample controls without DPPH working solution and a blank control without prodigiosin were prepared in parallel.

The DPPH radical scavenging activity was calculated as follows:

$$DPPH\;radical\;scavenging\;rate\;\left(D\%\right)=\frac{Ab-As+Ac}{Ab}\times 100\%$$

where Ab is the absorbance of the blank control, As is the absorbance of the sample reaction mixture, and Ac is the absorbance of the sample control.

### 2.7. Statistical Analysis

Unless otherwise stated, experiments were performed in triplicate, and data were expressed as mean ± standard deviation. The BBD regression model and ANOVA were performed using Design-Expert 13 software. Differences among treatment groups in stability, antibacterial activity, and DPPH radical scavenging activity should be analyzed using an appropriate statistical test, with *p* < 0.05 considered statistically significant.

## 3. Results and Discussion

### 3.1. Isolation and Identification of Serratia marcescens BD2025

**Figure 1:**
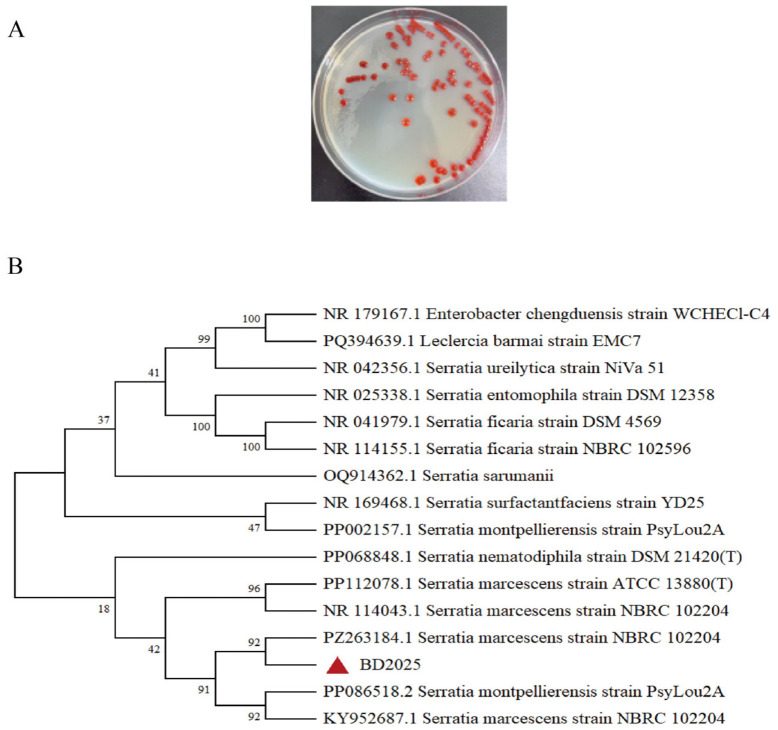
Identification of *Serratia marcescens* BD2025. (**A**) Colony morphology of strain BD2025 on NA medium. (**B**) Phylogenetic tree based on 16S rRNA gene sequence analysis.

After serial dilution of the naturally fermented soybean residue suspension and incubation on NA plates at 28 °C for 48 h, colonies with different morphologies were obtained. A red-pigmented colony was selected and purified by repeated streaking. As shown in Figure 1A, the purified isolate formed bright red, circular, convex colonies with smooth surfaces and entire margins on NA medium. The isolate was named BD2025.

The taxonomic identity of strain BD2025 was further determined by 16S rDNA gene sequencing. Phylogenetic analysis showed that BD2025 clustered with *Serratia marcescens* strain NBRC 102204 (Figure 1B). Therefore, this isolate was identified as *Serratia marcescens* BD2025 and used for subsequent prodigiosin production.

### 3.2. Extraction and Characterization of Prodigiosin

**Figure 2:**
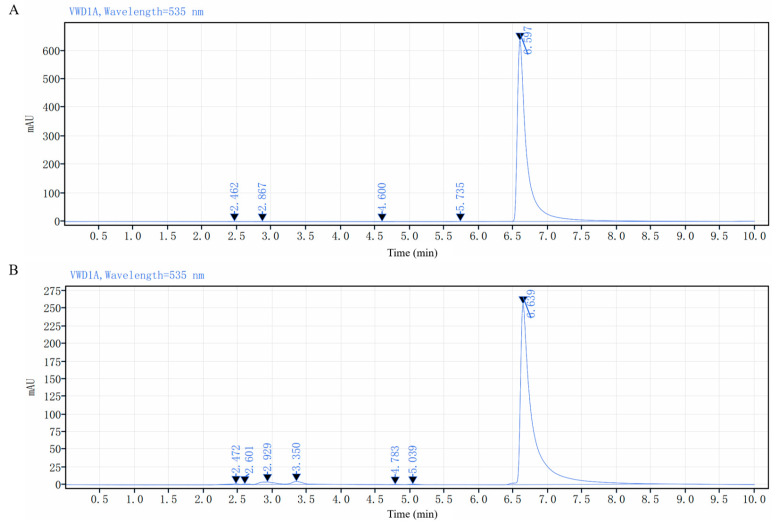
HPLC analysis of prodigiosin. (**A**) Prodigiosin standard. (**B**) Ethanol-extracted pigment produced by *S. marcescens* BD2025 during solid-state fermentation of soybean residue.

The pigment produced by *S. marcescens* BD2025 during solid-state fermentation of soybean residue was extracted with ethanol and analyzed by HPLC. As shown in Figure 2A, the prodigiosin standard showed a major peak at a retention time of 6.597 min at 535 nm. The ethanol-extracted pigment showed a major peak at 6.639 min under the same chromatographic conditions (Figure 2B). The close agreement in retention time between the sample and the standard indicates that the extracted pigment was consistent with prodigiosin. The relative purity of the extracted prodigiosin was estimated to be 96% using the peak area normalization method. This result indicates that the pigment extracted in this study was prodigiosin, and the purity was 96%.

### 3.3. Optimization of Prodigiosin Production Using a Box–Behnken Design

The production of prodigiosin is affected by multiple external factors. Previous studies have shown that the fermentation substrate and environmental factors, including moisture content, temperature and pH, significantly affect the production of prodigiosin [34]. Prodigiosin is generally synthesized by *S. marcescens* as a secondary metabolite during the late growth phase; therefore, an adequate fermentation period is essential for pigment accumulation [22]. Previous studies have reported that prodigiosin production by *S. marcescens* generally occurs within 15–84 h, depending on the strain and cultivation conditions [35,36]. Based on preliminary experiments, a fermentation time of 48 h was selected for *S. marcescens* BD2025.

To reduce the production cost of prodigiosin and increase the yield, in this study, soybean residue was used as the fermentation medium for *S. marcescens* BD2025. To improve prodigiosin production from soybean residue, three fermentation parameters were optimized using response surface methodology with a Box–Behnken design: soybean residue-to-water ratio, inoculum size, and temperature. The experimental design and observed prodigiosin yields are shown in Table 2. Among the experimental runs, the highest observed yield was 19.49 mg/g under the center-point conditions, corresponding to a soybean residue-to-water ratio of 1:4, an inoculation size of 5%, and a temperature of 28 °C. These results indicate that moderate water addition, inoculum size, and temperature were favorable for prodigiosin accumulation during solid-state fermentation.

The analysis of variance for the fitted regression models is shown in Table 3. The overall model was highly significant, with a model *p*-value < 0.0001, whereas the lack of fit was not significant (*p* > 0.05). The coefficient of determination values were high, with R^2^ = 0.9965, adjusted R^2^ = 0.9919, and predicted R^2^ = 0.9918, indicating good agreement between the experimental and model-predicted values. The linear effects of soybean residue-to-water ratio, inoculum size, and temperature were significant. The interaction terms between soybean residue-to-water ratio and inoculum size, and between soybean residue-to-water ratio and temperature, were also significant, whereas the interaction between inoculum size and temperature was not significant. The significant quadratic terms further indicate that prodigiosin production responded nonlinearly to the selected variables.

Based on the established model, the regression equation is as follows:

Y = 12.0663 + 5.91328 X1+ 1.54426 X2 − 2.99513 X3 − 0.37915 X1 X2 + 0.673263 X1 X3 + 0.272275 X2 X3 − 1.27055 X1^2^ − 3.00931 X2^2^ − 7.38794 X3^2^

where Y represents the predicted prodigiosin yield (mg/g), and X1, X2, and X3 respectively represent the ratio of soybean residue to water, inoculum size, and temperature.

**Figure 3:**
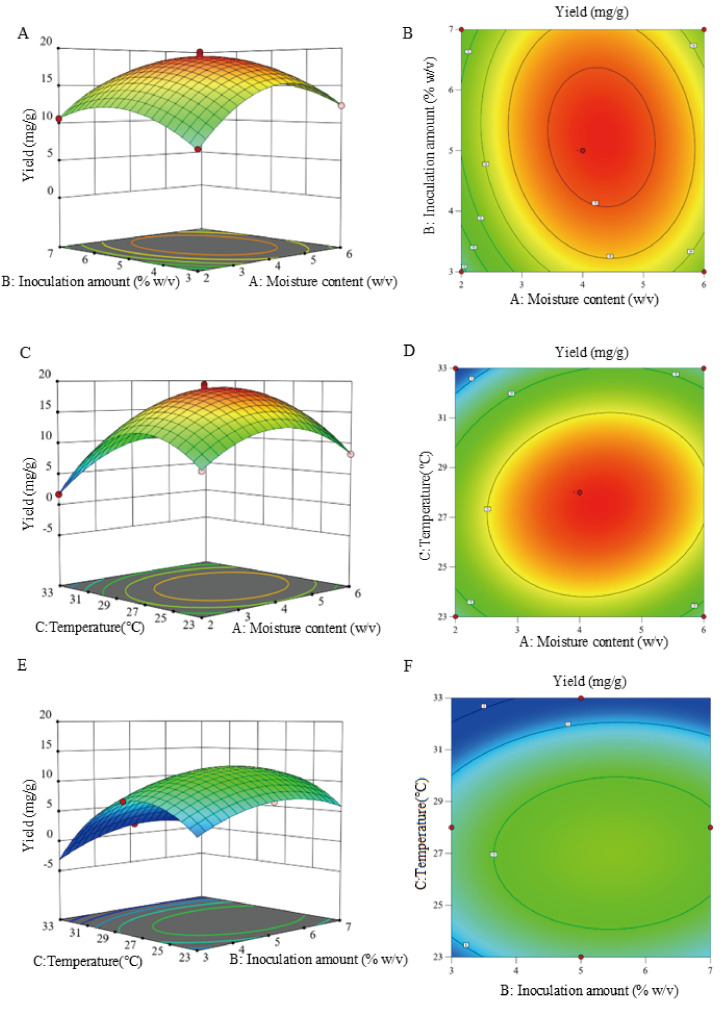
Effect of different variables on prodigiosin production, presented as a 3D plot (**left**) and contour plot (**right**). (**A**) 3D plot—the ratio of soybean residue to water with the inoculation size. (**B**) Contour plot—the ratio of bean residue to water with the inoculation size. (**C**) 3D plot—the ratio of bean residue to water with temperature. (**D**) Contour plot—the ratio of bean residue to water with temperature. (**E**) 3D plot—inoculation size with temperature. (**F**) Contour plot—inoculation size with temperature. The color gradient from blue to red represents the predicted yield, with warmer colors indicating higher production.

The 3D response surface and contour plots further illustrate the effects of the selected variables on prodigiosin production (Figure 3). The model predicted an optimal soybean residue-to-water ratio of 1:4.28, an inoculum size of 5.2%, and a cultivation temperature of 27.5 °C, with a predicted prodigiosin yield of 19.05 mg/g. The result is significantly higher than what has been reported in similar studies. For example, solid-state fermentation with *Serratia marcescens* on soybean dreg, rice bran and fish meal substrates yielded only 79, 50 and 54 mg/kg, respectively [19,20,21,22]. The yield of solid-state fermentation on LB1 agar plates was also only 22 mg/kg [22]. In contrast, the yield in this study is about several hundred times that of the solid-state fermentation described above. This fully demonstrates the high-yield potential of *S. marcescens* BD2025 in the production of prodigiosin, and it also verifies the effectiveness of the Box–Behnken design in optimizing fermentation conditions. In conclusion, this study not only has significant advantages in substrate selection and process optimization, but also provides a solid technical foundation for the high-value utilization of soybean residue and the green industrial production of prodigiosin.

Moisture content is a critical factor in solid-state fermentation. Insufficient water limits nutrient diffusion and microbial metabolic activity, whereas excessive water can reduce substrate porosity and oxygen transfer, making the system closer to submerged fermentation. Kuo and Li et al. reported that prodigiosin production from rice bran and fish meal was strongly influenced by water addition and that excessive moisture was strongly influenced by water addition and unfavorable for pigment accumulation [22]. The present results are consistent with this observation, as prodigiosin yield increased at moderate soybean residue-to-water ratios but declined under less favorable hydration conditions.

The inoculation size is regarded as an important factor in the solid-state fermentation process. An insufficient inoculation dose results in a low initial bacterial concentration, which in turn affects the pigment production. In previous studies, the optimal inoculation dose for prodigiosin production using bioreactors was 8 to 10% [37]. Our previous single-factor experiments results showed that the production of prodigiosin first increased and then decreased with the increase in inoculum, indicating that the production of prodigiosin was not utilized when the inoculum was too high. Some studies have suggested that due to the complex relationship between population sensing and the production of secondary metabolites, a high initial bacterial density has an adverse effect on the production of secondary metabolites in solid fermentation substrates [38]. However, the Petri dish used in this study was too small, and the optimal inoculation volume differed somewhat from that of the bioreactor.

Temperature is another key determinant of prodigiosin production [34]. It is reported that the production of prodigiosin by *Serratia* will be affected when the temperature exceeds 30 °C. The reason is that above 30 °C, the condensing enzyme *Pig C*, which is the final step in the synthesis of prodigiosin, will denature, making it difficult to synthesize prodigiosin [34]. Therefore, the optimum temperature for prodigiosin production is usually 22 to 30 °C, which is related to the species characteristics, and the optimum temperature for BD2025 in this study was 27.5 °C, which is within the optimum temperature range of *Serratia*. The present results therefore support the need for temperature control during soybean residue-based solid-state fermentation by *S. marcescens* BD2025.

### 3.4. Stability of Prodigiosin

**Figure 4:**
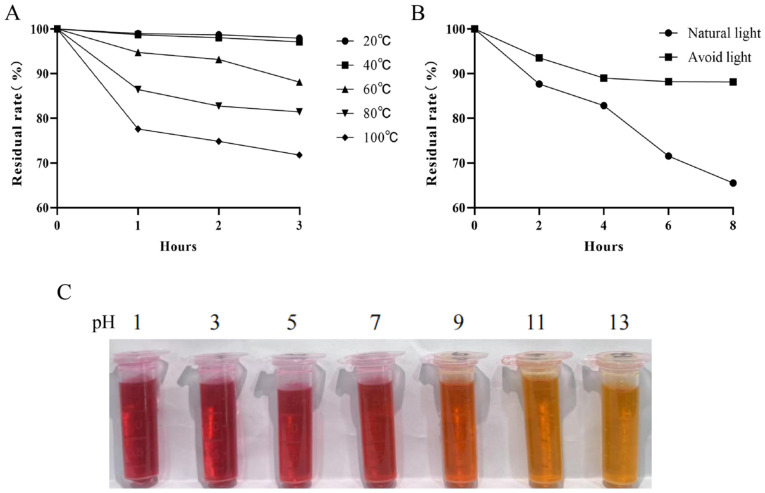
Stability of prodigiosin under different environmental conditions. (**A**) Temperature stability. (**B**) Light stability. (**C**) Color change under different pH conditions.

The stability of prodigiosin under different environmental conditions was evaluated by monitoring the residual absorbance at 535 nm. As shown in Figure 4A, prodigiosin remained relatively stable at 20 °C and 40 °C. However, pigment stability decreased markedly when the temperature exceeded 60 °C, indicating that elevated temperature accelerated pigment degradation. This thermal instability may be associated with disruption of the conjugated pyrrole system or structural changes in the monopyrrole and dipyrrole moieties of prodigiosin [39].

Light exposure also influenced prodigiosin stability. As shown in Figure 4B, with the passage of time, the OD535 nm value of prodigiosin under dark conditions remained basically unchanged, and the retention rate was still above 90%. However, under natural light conditions, the OD535 nm value of prodigiosin showed a downward trend, and the retention rate of the pigment was 65.51% after 8 h. The results show that the stability of prodigiosin under natural light was significantly different from that in dark environment. Therefore, light exposure has a considerable impact on prodigiosin. In the presence of light, these pigment molecules are in an excited state, which helps to enhance their reaction capabilities [40]. This reaction led to the disappearance of the color, because the pyrrole group in the lichen red pigment underwent delocalization [41].

The pH stability results show that prodigiosin was more stable under acidic and neutral conditions than under alkaline conditions. In the acidic range, the pigment solution is red; when the pH is greater than or equal to 9, the pigment solution gradually changes from red to orange-yellow (Figure 4C). As shown in Table 4, this red pigment is relatively stable in acidic solutions. When the pH is between 1 and 7, its residual rate remains above 87%, and there is a slight change in color, with the redness becoming weaker. Between pH 9 and 13, the color turns into an orange-yellow shade, significantly fading, and the residual rate is less than 50% at pH 13. These color changes are consistent with previous studies [18]. This may be due to the fact that under extremely acidic conditions, the pyrrole group is protonated on the second carbon atom of the ring, rather than on the nitrogen atom, and thus is no longer aromatic [42]. In an extremely alkaline environment, the -OH group will lose the hydrogen atom in the amine structure, thereby forming an anion [42]. In both cases, the conjugated system formed by the double bond is destroyed, leading to degradation of the pigment [43]. Overall, the extracted prodigiosin showed better stability at 20–40 °C.

### 3.5. Antibacterial Activity of Prodigiosin

**Figure 5:**
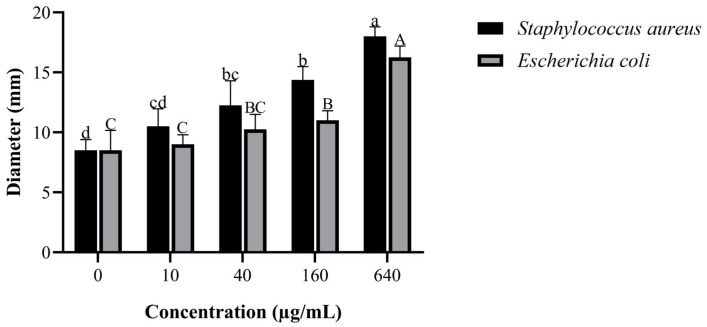
Antibacterial activity of prodigiosin against *E. coli* and *S. aureus.* Different capital letters indicate significant differences (*p* < 0.05) in the effect of prodigiosin at different concentrations on *Escherichia coli*, while the same letters indicate no significant difference (*p* > 0.05). Different lowercase letters indicate significant differences (*p* < 0.05) in the effect of prodigiosinat different concentrations on *Staphylococcus aureus*, while the same letters represent no significant differences (*p* > 0.05).

The antibacterial activity of prodigiosin was evaluated against *E. coli* and *S. aureus* using the agar disk diffusion assay. As shown in Figure 5, prodigiosin inhibited both bacterial strains, and the inhibition-zone diameter increased with increasing prodigiosin concentration. These results indicate that the antibacterial activity of prodigiosin was concentration-dependent within the tested range.

The inhibition zone against *S. aureus* were larger than those against *E. coli* at the same prodigiosin concentrations, suggesting that *S. aureus* was more susceptible to the extracted prodigiosin. This observation is consistent with previous reports showing that prodigiosin exhibits broad-spectrum antibacterial activity against both Gram-positive and Gram-negative bacteria, with stronger inhibitory effects often observed against Gram-positive bacteria [44,45].

The difference in susceptibility between *S. aureus* and *E. coli* may be associated with differences in extracellular membrane structure. This might be due to the fact that *S. aureus* (Gram-positive bacteria) lacks an outer membrane structure, and its cell wall is composed only of peptidoglycan, through which prodigiosin can freely penetrate [46]. The lipopolysaccharide structure on the outer polysaccharide layer of Gram-negative bacteria is impermeable to lipophilic solutes. Gram-negative bacteria are more difficult to be penetrated by the lipophilic prodigiosin than Gram-positive bacteria [46]. These results suggest that prodigiosin produced from soybean residue-based fermentation has potential antimicrobial activity.

### 3.6. Antioxidant Activity of Prodigiosin

**Figure 6:**
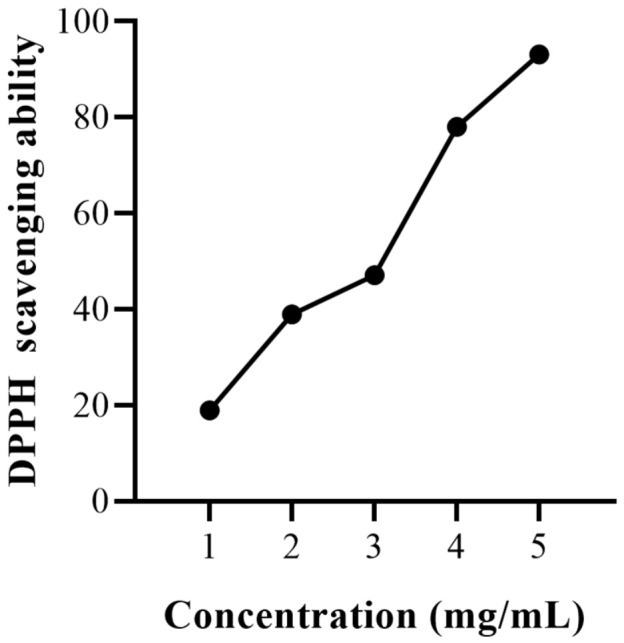
DPPH radical scavenging activity of prodigiosin at different concentrations.

The antioxidant activity of prodigiosin was evaluated using the DPPH radical scavenging assay. As shown in Figure 6, prodigiosin exhibited concentration-dependent radical scavenging activity. At concentrations of 1, 2, 3, 4 and 5 mg/mL, the DPPH radical scavenging activities were 18.94%, 38.96%, 47.15%, 78.00% and 93.13%, respectively.

The DPPH radical scavenging activity of prodigiosin may be attributed to its tripyrrole ring structure and conjugated system, which can participate in electron or hydrogen atom transfer and thereby stabilize free radicals [33]. Previous studies have also reported that the radical scavenging ability of prodigiosin is dependent on both concentration and reaction time, with higher concentrations and longer reaction times generally leading to stronger scavenging effects [33]. The present results indicate that prodigiosin produced by *S. marcescens* BD2025 using soybean residue as the fermentation substrate has in vitro antioxidant potential. However, because the DPPH assay represents a chemical radical scavenging system, additional antioxidant assays would be required to evaluate its activity in more complex biological or food-related systems.

## 4. Conclusions

In this study, a strain of *Serratia marcescens* BD2025 with a high yield of prodigiosin was isolated from naturally fermented soybean residue. Through Box–Behnken design optimization, under the conditions of a bean residue-to-water ratio of 1:4.28, an inoculum size of 5.2%, and a cultivation temperature of 27.5 °C for 48 h, the maximum yield of prodigiosin reached 19.05 mg/g. The property study showed that this pigment has good stability under dark, low-temperature and acidic conditions. It exhibited significant antibacterial activity against both *Escherichia coli* and *Staphylococcus aureus*, and its DPPH radical scavenging ability showed a dose-dependent relationship. Overall, these findings indicate that soybean residue can be used as a low-cost substrate for solid-state prodigiosin production, providing a potential value-added route for soybean-processing waste utilization. Further work should focus on process scale-up, yield validation under optimized conditions, and comprehensive evaluation of product safety and application performance.

## Figures and Tables

**Table 1:** The levels and ranges of the variables in the BBD.

Variable Factors	Coded Symbol	Coded Levels		
		−1	0	1
Soybean residue-to-water ratio (*w*/*v*)	X1	1:2	1:4	1:6
Inoculation size (% *w*/*v*)	X2	3	5	7
Temperature (°C)	X3	23	28	33

**Table 2:** Experimental design matrix and prodigiosin yield.

Run	Experimental Variables			Prodigiosin Yield (mg/g)
	X1	X2	X3	
1	1	1	0	12.3075
2	−1	1	0	10.6115
3	−1	0	−1	7.5428
4	0	1	1	7.7938
5	−1	−1	0	7.6142
6	0	−1	−1	9.5776
7	−1	0	1	1.7023
8	0	0	0	17.8903
9	0	0	0	19.2101
10	0	−1	1	5.5861
11	0	0	0	18.9686
12	0	0	0	18.4922
13	0	1	−1	10.6962
14	0	0	0	19.4922
15	1	0	−1	8.2857
16	1	0	1	7.8313
17	1	−1	0	12.3434

**Table 3:** ANOVA of regression models for optimizing prodigiosin production.

Source	Sum of Squares	df	Mean Square	*F*-Value	*p*-Value
Model	472.88	9	52.54	218.42	<0.0001
A—Soybean residue-to-water ratio	130.11	1	130.11	540.86	<0.0001
B—Inoculation size	6.36	1	6.36	26.44	0.0013
C—Temperature	23.92	1	23.92	99.44	<0.0001
AB	2.30	1	2.30	9.56	0.0175
AC	7.52	1	7.25	30.15	0.0009
BC	0.2965	1	0.2965	1.23	0.3036
A^2^	108.75	1	108.75	452.09	<0.0001
B^2^	38.13	1	38.13	158.51	<0.0001
C^2^	229.82	1	229.82	955.35	<0.0001
Residual	1.68	7	0.2406		
Lack of fit	0.0864	3	0.0288	0.0721	0.9718
Pure error	1.60	4	0.3994		
Cor Total	474.57	16			

R^2^ = 99.65%; R^2^(adj) = 99.19%; R^2^(pred) = 99.18%.

**Table 4:** pH stability of prodigiosin after 4 h of treatment.

pH	0 h	4 h	Residual Rate (%)
1	1.333	1.233	92.5 ^a^
3	1.270	1.153	90.79 ^b^
5	1.253	1.104	88.11 ^c^
7	1.241	1.085	87.43 ^d^
9	1.218	0.888	72.91 ^e^
11	0.868	0.478	55.07 ^f^
13	0.665	0.284	42.71 ^g^

Values with different lowercase letters indicate significant differences among pH treatments (*p* < 0.05).

## Data Availability

The original contributions presented in this study are included in the article. Further inquiries can be directed to the corresponding author.
